# Relationships between extended high frequency hearing, cognition, and speech-in-noise performance

**DOI:** 10.3389/fnins.2026.1933436

**Published:** 2026-10-20

**Authors:** Claire M. Dorey, David A. Eddins, Ann C. Eddins, Nathan C. Higgins, Carrie A. Secor, Victoria A. Sanchez, Erol J. Ozmeral

**Affiliations:** 1 Department of Communication Sciences and Disorders, College of Behavioral and Community Sciences, University of South Florida, Tampa, FL, United States; 2 School of Communication Sciences and Disorders, College of Health Professions and Sciences, University of Central Florida, Orlando, FL, United States; 3 Department of Otolaryngology, Audiology Section, Morsani College of Medicine, University of South Florida, Tampa, FL, United States

**Keywords:** aging, audiology, dementia, functional performance, hidden hearing loss, MoCA = Montreal cognitive assessment, sex diferences, words in noise (WIN)

## Abstract

**Introduction:**

Impaired speech-in-noise performance is a primary patient complaint in audiology clinics, and in addition to standard frequency (SF; 0.25–8 kHz) sensitivity, age, and cognition, recent work as focused on the association of extended high frequency (EHF, > 8 kHz) sensitivity with this degraded performance. Despite this accepted relationship, clinical utility of EHFs is limited because there is not a universally accepted cutoff value for normal vs. impaired sensitivity. The objective of the current study was to analyze the relationship between EHF sensitivity, SF sensitivity, cognition, and age with speech in-noise performance, as measured by the Words-in-Noise (WIN) test.

**Methods:**

The SF audiometry, EHF audiometry, and speechin-noise assessments were measured in 605 listeners between the years 2015 and 2024. Pure tone average (PTA) for both the SF (PTA_SF_: 0.5, 1, 2, & 4 kHz) and EHF (PTA_EHF_: 9, 10, 12.5, & 14 kHz) were calculated for each participant. A normal hearing (NH) group was defined by having a PTA_SF_ ≤ 20 dB HL (*N* = 700 ears) and a hearing loss group (HL) had a PTA_SF_ > 20 dB HL (*N* = 505 ears). Cognition was also measured via the Montreal Cognitive Assessment (MoCA) in 524 of the participants.

**Results:**

PTA_SF_ and PTA_EHF_ were both found to be associated with WIN performance across all participants. However, when we analyzed solely participants with NH, PTA_EHF_ explained more variance in WIN thresholds than PTA_SF_. Data were further assessed to determine functional cutoffs. The NH participants were broken into 5-dB-HL bins to determine cutoff values for WIN performance, and significant cutoffs were found at a PTA_SF_ > 10 dB HL and a PTA_EHF_ > 35 dB HL. The EHF_PTA_, MoCA scores, and age each had a significant effect on WIN threshold, but EHF_PTA_ was found to be a stronger predictor, despite limited EHF spectral information in the WIN stimuli.

**Discussion:**

These findings are consistent with previous literature and support the clinical relevance of EHF sensitivity. EHF threshold measurements may provide valuable information, given their association with speech-in-noise deficits, a common patient complaint.

## Introduction

Growing evidence suggests that poorer extended high frequency (EHF) hearing detection thresholds (at frequencies > 8 kHz) are associated with the degree of self-reported hearing loss, warranting clinical inclusion in a standard test battery (Helfer et al., 2024; Motlagh Zadeh et al., 2019). However, clinical application has been limited in part because the field has yet to reach a universal definition of “normal hearing” in the EHF range, despite discussions in the literature establishing that an EHF cutoff value is feasible and recommended (Rodríguez Valiente et al., 2014; Shahnaz and Ciocca, 2026; Stenklev and Laukli, 2004).

Cutoff thresholds for the standard frequency (SF; 0.25–8 kHz) in adults have a complex history and there has been international disagreement on the dividing line. Guidelines were once derived from standards produced by the International Organization for Standardization (ISO); however, for economic reasons, cutoff values were ultimately shifted upwards in the United States to limit the percentage of the population determined to have a hearing impairment (Gatlin and Dhar, 2021). More recently, the World Health Organization (WHO) also convened a panel of experts to determine cutoff criteria and changed their recommendation for normal hearing in the SF range to a pure tone average (PTA) of ≤20 dB HL (Humes, 2019). Importantly, neither the ISO or WHO methods of determining “normal” hearing are centered on functional, or real world, performance, as has been typical for pediatric guidelines. The pediatric cutoff for normal hearing was determined to be 15 dB HL through parental perceived deficit, educational performance in children with “minimal” hearing loss, and effects of threshold elevation to 20 dB on speech perception, specifically how distortion of high frequency consonant sounds could affect performance (Northern and Downs, 2002). Functional measures are paramount to the discussion of cutoff values for normal hearing and should be considered in addition to context of typical thresholds by age.

Decreased speech-in-noise performance, a functional and supra-threshold deficit, is a key complaint of many individuals with hearing loss but has also been seen in populations with normal hearing (Mishra et al., 2022b; Motlagh Zadeh et al., 2019). Previous work has found EHF thresholds to be correlated with speech-in-noise performance in both adult and pediatric populations (Hunter et al., 2020; Mishra et al., 2022b; Monson and Buss, 2022; Motlagh Zadeh et al., 2019). Therefore, EHF threshold measurement may provide clinical utility in diagnostic audiology.

Of particular interest are the EHF hearing thresholds in individuals with normal SF hearing thresholds. This phenomenon has been often reported in research and is not an uncommon clinical complaint (Bharadwaj and Shinn-Cunningham, 2021; Kohrman et al., 2020; Tremblay et al., 2015; Valderrama et al., 2022). Normal hearing at SF with self-perceived difficulty has been coined “hidden hearing loss” (Schaette and McAlpine, 2011), and elevated EHF thresholds may provide some clarity in the phenomenon and possibly one mechanistic explanation, i.e., cochlear synaptopathy (Kujawa and Liberman, 2009; Liberman et al., 2016).

Our previous work showed a correlation between PTA at the SF (PTA_SF_: 0.5, 1, 2, & 4 kHz) and EHF (PTA_EHF_: 9, 10, 12.5, & 14 kHz) ranges (Dorey et al., 2026). However, at the individual level, EHF hearing thresholds can be quite different in patients with similar, normal SF hearing thresholds, and poor EHF thresholds do not solely occur in populations with poor SF thresholds. This individual variability may contribute to perceived functional deficits and measurable differences in performance among individuals with similar, normal standard audiometric thresholds.

While EHF hearing has been linked to variability in speech-in-noise performance regardless of hearing status, other confounding factors to consider are effects of aging and cognition. Kocabay et al. (2022) reported age, hearing loss, and cognition all contribute to speech-in-noise performance, with age and hearing loss explaining the greatest amount of variance. However, this work explored only the SF range, not EHF hearing. A recent analysis of 347 adults from Helfer et al. (2024) discussed the relationship between EHF hearing and (1) demographics, risk factors for hearing loss, and cognitive decline; and (2) speech-in-noise performance. The association between EHF thresholds and cognition yielded varying results dependent on age, but degraded speech-in-noise performance associated with higher EHF thresholds was observed regardless of participant age (Helfer et al., 2024). It is important to note, however, that cognition is a complex covariant in relation to hearing loss. A large cross-sectional study using the UK Biobank found that comorbidities, including dementia, are prevalent in around 13% of the population with hearing loss (Yévenes-Briones et al., 2024), and cognitive decline is heavily correlated with hearing loss across the literature (Chern and Golub, 2019; Goll et al., 2011; Gurgel et al., 2014; Marinelli et al., n.d.; Qian et al., 2017; Uhlmann et al., 1989). Hearing loss has also been cited consistently as a predominant potentially modifiable risk factor for cognitive decline (Livingston et al., 2024; Livingston et al., 2020; Livingston et al., 2017; Loughrey et al., 2018), but these associations have been established strictly in relation to elevation of SF hearing, not inclusive of EHF hearing thresholds.

In this study, we present clinically relevant functional and EHF audiometric data to provide a clearer basis for establishing a clinical cutoff value of EHF hearing thresholds. The objective of the current study was to retrospectively explore the relationship between EHF hearing and speech-in-noise performance, and a secondary goal was to analyze cognition and aging alongside EHF hearing sensitivity as predictors of speech-in-noise performance.

## Methods

### Design and participants

This was a retrospective re-analysis of laboratory recruited participants. Participants were selected for inclusion if they were ≥18 years of age and their audiometric assessment included EHF thresholds. An air-bone gap ≥15 dB at any frequency 0.25, 0.5, 1, 2, or 4 kHz was considered exclusion criteria. All methodology was performed in compliance with the University of South Florida’s Institutional Review Board (#STUDY002799, #STUDY000265, #STUDY008457).

Participants were 605 adults (male *N* = 246; female *N* = 359) between the ages of 18 and 88 years old (mean = 48.82 years, SD = 22.94 years; Figure 1) who were recruited at the University of South Florida in the Auditory Neurosciences and Technology Laboratory (formerly the Auditory & Speech Sciences Laboratory) and the Auditory Rehabilitation and Clinical Trials Laboratory between the years 2015 and 2024. Participants self-identified race and ethnicity based on U. S. Census guidelines (Table 1).

**Figure 1:**
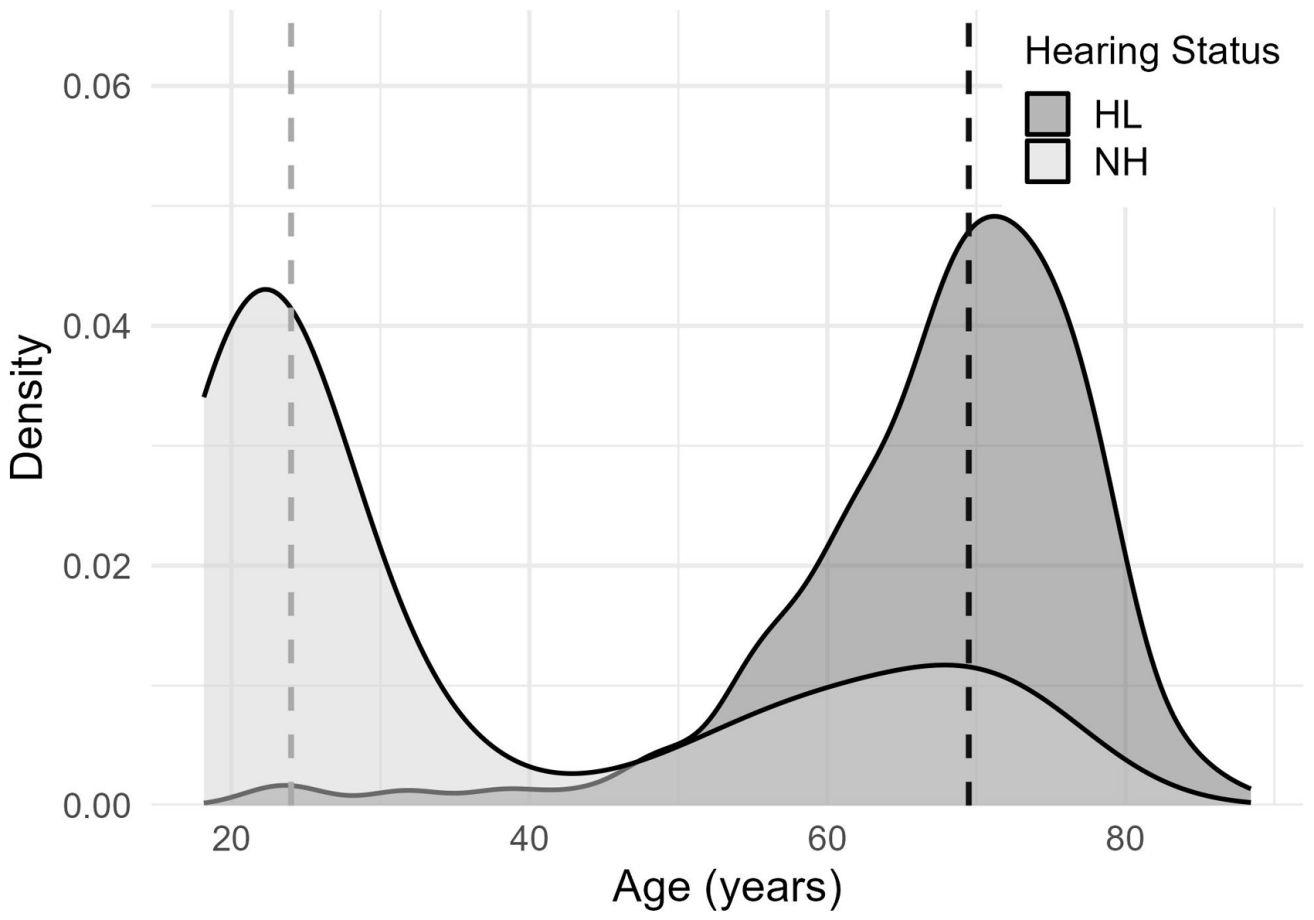
Distribution of hearing status by age for both the NH (PTA_SF_ ≤ 20 dB HL) and HL (PTA_SF_ > 20 dB HL) groups. Participant ages were 18 to 88 years old (NH, normal hearing; PTA_SF_, standard frequency pure tone average; HL, hearing loss).

**Table 1:** Demographic information for all 605 participants.

Demographics	*N*
Race	
American Indian/Alaska Native	2
Asian	38
Black or African American	39
More than One Race	4
White	505
Unknown/Not Reported	17
Total	605
Ethnicity	
Hispanic or Latino	66
Not Hispanic or Latino	527
Unknown/Not Reported	12
Total	605

Participants were asked to self-report race and ethnicity based on U. S. Census classifications.

### Audiometry

SF audiometry (0.25–8 kHz) and EHF audiometry (9–16 kHz) were measured either by licensed audiologists or Doctor of Audiology students under the supervision of a licensed audiologist using the GSI 61, Audiostar or Audiostar Pro audiometers (Grayson-Stradler Inc.; Prairie, MN) and the modified Huston Westlake procedure (Carhart and Jerger James, 1959). Audiometry was conducted in double-walled sound attenuation booths using Radio Ear DD 450 and Sennheiser HDA-200 circumaural headphones which have been found to have transferable reference equivalent threshold sound pressure levels (RETSPLs; Smull et al., 2019). All audiometers were calibrated annually in compliance with ANSI S3.6 (American National Standards Institute, 2025). “No Response” thresholds were reported as 5 dB HL above the audiometer output limits. Audiometer output limits and reported level for No Response thresholds are shown in Supplementary Table 1. Pure tone averages (PTA) were calculated for both the SF (PTA_SF_; 0.5, 1, 2, & 4 kHz) and EHF (PTA_EHF_; 9, 10, 12.5, & 14 kHz) ranges. Though there is no agreed upon set of frequencies included in the PTA_EHF_, the frequencies included in here align with our previous work (Dorey et al., 2026), and a discussion regarding the selected frequencies for PTA_EHF_ can be found there. In addition, due to the nature of this work being retrospective, only ears with complete audiometric evaluations in both the SF and EHF ranges were included. Because of this rule and our exclusion criteria, 5 participants only had inclusion of one ear, bringing the total ears evaluated to 1,205.

### Speech-in-Noise

The Words-in-Noise (WIN; Wilson, 2003) test was administered using the same equipment as audiometry and the standard testing procedures outlined in Wilson (2011). This test is especially relevant due to the recent recommendation by the National Academies for Sciences, Engineering, and Medicine (NASEM) of the WIN as a primary outcome measure in audiology (National Academies of Sciences, Engineering, and Medicine, 2025). The WIN is a monaural word recognition test that presents five Northwestern University Auditory Test No. 6 (NU-6) words, spoken by a female speaker in multi-talker babble background noise. The presentation level of the multi-talker babble was fixed, and the words were presented first at a louder level (easier SNR) then reduced every five words, from 24 to 0 dB SNR in 4 dB steps (Wilson et al., 2003). A 35-word list was presented. Participants repeated back the word they heard and the threshold was determined as the SNR at which word recognition performance was 50%, as determined by the Spearman-Karber equation (Spearman, 1908; Wilson, 2011), resulting in a threshold interpretation between −2.0 and 26 dB SNR. A higher threshold here is indicative of poorer performance. Participants were not provided a practice in advance of testing.

With participant recruitment occurring between two separate laboratories and three IRB protocols, there were different speech-in-noise protocols due to primary study goals and study populations. Because the target speech and babble are presented in the same channel, the presentation level refers to the peak speech level + babble at 24 dB SNR. In the Auditory Neurosciences and Technology Laboratory, the WIN test was administered at varying presentation levels depending on a participant’s hearing status. The presentation level varied according to the following criteria (1) 80 dB HL if the participant’s monaural 3-frequency PTA (0.5, 1, 2 kHz) was ≤40 dB HL; (2) 90 dB HL if the participant’s monaural 3-frequency PTA was between 40 and 59 dB HL; and otherwise (3) words in quiet without babble only at either 90 dB HL or their most comfortable level (MCL) if the PTA exceeded 60 dB HL. Alternatively, in the Auditory Rehabilitation and Clinical Trials Laboratory, the WIN was presented at both 40 and 70 dB HL regardless of hearing status because study questions revolved around potential level differences. We retained WIN thresholds obtained at 40, 70, 80, and 90 dB HL in our dataset. The WIN thresholds presented at the 70 dB HL level from the Auditory Rehabilitation and Clinical Trials lab were selected for primary analysis in conjunction with data from the Auditory Neurosciences and Technology Laboratory. The WIN is administered using an ear-specific normative cutoff of 6-dB SNR, with thresholds greater than 6 dB SNR indicating poorer speech-in-noise performance (Wilson, 2011).

### Cognitive assessment

The Montreal Cognitive Assessment (MoCA; version 7.1; Nasreddine et al., 2005) was administered to participants using pen and paper by trained staff on paper. The MoCA is a widely used outpatient cognitive screener in which a score of 26 or higher is associated with normal cognitive function. MoCA instructions are verbally given to the participant, making it a binaural task; therefore, the proceeding MoCA analysis was performed by participant, not by ear.

*Analysis:* Statistical analysis was performed in R (R Core Team, 2025) using *tibble, dplyr, ggplot2, broom, lme4*, *lmerTest, and partR2* packages. Because the WIN is a monaural task, all analysis unless otherwise reported is discussed as number of ears, not participants. Due to the non-independence of ears with participants, a linear mixed effects (LME) model with a random intercept for participant was used to assess the majority of our research questions. These models produce a marginal *R^2^* value to explain the variance of the fixed effects and a conditional *R*^2^ representative of the variance explained by the combination of fixed and random effects.

The LME model was run to assess contributions of PTA_SF_ and PTA_EHF_ on WIN performance in all participants, and then again in only participants with normal hearing. To determine if either PTA_SF_ or PTA_EHF_ was a greater predictor of WIN performance, semi-partial *R*^2^ values for each fixed effect were calculated. Based on 1,000 bootstrap resamples, marginal *R*^2^ was used to determine the variance unique to each predictor and the variance shared between predictors (Stoffel et al., 2021).

The PTA_SF_ and PTA_EHF_ values were categorized into 5-dB bins to evaluate whether WIN performance differed from the 6-dB-SNR normative criterion (Wilson, 2011). Within each PTA_SF_ bin, the association between WIN performance and PTA_EHF_ was examined using separate LME regression models with a random intercept for participant. Next, to identify potential clinically relevant thresholds within the SF and EHF ranges, separate LME models were conducted for each bin, with the fixed-effect intercept tested against the 6-dB SNR criterion using a two-tailed test.

To further characterize the relationship between hearing sensitivity and extremes of WIN performance in the NH population, the 10% of participants with the best and worst WIN thresholds were identified, and their WIN performance was examined in relation to their PTA_EHF_ using LME models with a random intercept for participant to account for the inclusion of both ears from most participants. Finally, effects of sensation level (SL) on the WIN were evaluated using a similar model because individuals received various presentation levels (as described above) and overall audibility was determined by their individual PTAs. In order to analyze if presentation level affects WIN performance, the SL for both the SF (SL_SF_) and EHF (SL_EHF_) ranges were calculated based on PTA_SF_ and PTA_EHF_ and the presentation level of the WIN. The SL was calculated as the difference between presentation level and PTA to determine the level of audibility of the presentation level relative to the participant’s hearing sensitivity. A LME model was run to assess WIN thresholds with SL_SF_ and SL_EHF_ as fixed effects with a random effect for participant.

Hearing sensitivity, cognition, and age were all evaluated as relative predictors of speech-in-noise performance. First, we evaluated the relative contributions of PTA_EHF_ and MoCA score on WIN performance. The MoCA is a binaural task, so PTA_EHF_ and WIN thresholds were averaged across ears. A multiple linear regression with semi-partial correlations was run with WIN thresholds as the outcome measure and PTA_EHF_ and MoCA score as predictors. For each predictor, the semi-partial correlation removed the variance shared with the other predictor before assessing the unique contribution to WIN threshold. Unstandardized coefficients are reported for both PTA_EHF_ and MoCA scores as raw units due to their clinical applicability. Next, we included age into our analysis. Relative contributions of PTA_EHF,_ MoCA score, and age on WIN performance were analyzed using a multiple linear regression on the averaged binaural data. All three predictors were standardized prior to analysis to allow for direct comparison of effect sizes across variables with differing units. Standardized regression coefficients were extracted to evaluate the relative contribution of each predictor on WIN performance while controlling for the other predictors in the model.

Lastly, sex differences in hearing sensitivity, cognition, and speech-in-noise performance were assessed using the same averaged data across ears. A one-way multivariate analysis of variance (MANOVA) was run with WIN performance, PTA_SF_, PTA_EHF_, and MoCA scores as dependent variables and sex as the between-subjects factor. Wilks’ λ was used as the multivariate test statistic. Significant effects were assessed with univariate ANOVAs for each dependent variable.

## Results

Because the WIN is a monaural task, all results in the following sections will be discussed as number of ears, not participants unless otherwise specified.

### Audiometry

Ears were broken into groups based upon hearing status. Aligning with the WHO recommendations for hearing loss classification (Humes, 2019), the normal hearing group (NH; PTA_SF_ ≤ 20 dB HL) included 700 ears. The hearing loss group (HL; PTA_SF_ > 20 dB HL) included 505 ears. The mean PTA_SF_ was 6.18 dB HL (SD = 5.56) for the NH group and 35.9 dB HL (SD = 11.6) for the HL group. The PTA_EHF_ for the NH group had a notable range of −10 dB HL to 95 dB HL (mean = 18.4 dB HL; SD = 27.2). For the HL group, PTA_EHF_ ranged from 0 to 97.5 dB HL (mean = 73.9 dB HL, SD = 16.2; Table 2).

**Table 2:** Pure tone averages (PTA) were calculated for both the standard frequency (SF) and extended high frequency (EHF) ranges.

Group	N (ears)	PTA_SF_				PTA_EHF_			
		Mean	Min	Max	SD	Mean	Min	Max	SD
NH (PTA_SF_ ≤ 20 dB HL)	700	6.18	−6.25	20	5.56	18.4	−10	95	27.2
HL (PTA_SF_ > 20 dB HL)	505	35.9	21.2	109	11.6	73.2	0	97.5	16.2

Participants were sorted into the normal hearing (NH) group if their PTA_SF_ was 20 dB HL or less. The hearing loss (HL) group was defined by PTA_SF_ of over 20 dB HL. EHF hearing was not reported as normal or abnormal.

### WIN performance and PTA

WIN thresholds ranged with interpretations from normal to profound speech-in-noise impairments with mean performance of 8.76 dB SNR (SD = 4.99) in our dataset (min = −2.0 dB SNR; max = 25.2 dB SNR). The results of the LME indicate that 66.6% of the variance in WIN thresholds was explained by both PTA_SF_ and PTA_EHF_ together in all ears (*N* = 1,205 ears in 605 participants) regardless of hearing status (marginal *R^2^* = 0.67; Figure 2). As shown in Figure 2, WIN thresholds were positively associated with both PTA_SF_ (*β* = 0.173, *t* = 17.91, *p* < 0.001) and PTA_EHF_ (*β* = 0.035, *t* = 7.47, *p* < 0.001). The same analysis was run in only the NH group (*N* = 700). Again, both PTA_SF_ (*β* = 0.066, *t* = 3.07, *p* = 0.002) and PTA_EHF_ (*β* = 0.05, *t* = 9.66, *p* < 0.001) were positively associated with WIN performance (marginal *R^2^* = 0.350, conditional *R^2^ =* 0.732; shown in Figure 3). To assess the relative contribution of each predictor, we calculated semi-partial *R*^2^ values. PTA_EHF_ (part *R^2^* = 0.17, 95% CI[0.104, 0.243]) had a greater independent influence on WIN performance in this population than PTA_SF_ (part *R*^2^ = 0.010, 95% CI [0, 0.094]).

**Figure 2:**
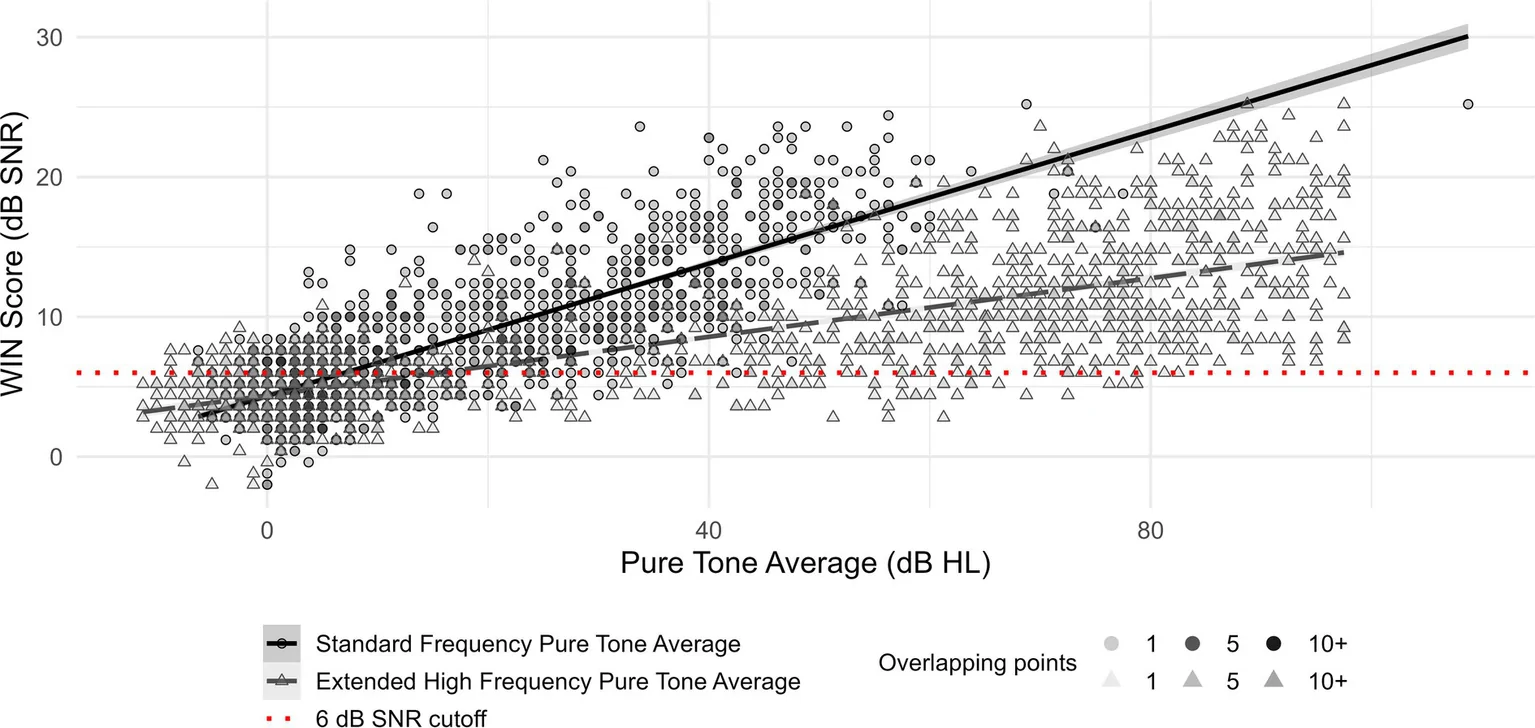
Words-in-noise (WIN) performance (dB SNR) as a function of standard frequency (PTA_SF_; 0.5, 1, 2, & 4 kHz) and extended high frequency (PTA_EHF_; 9, 10, 12.5, 14 kHz) PTA. Solid lines indicate linear fit for PTA_SF_ (circles) and PTA_EHF_ (triangles) and dotted red line indicates 6 dB SNR cutoff for normal speech-in-noise performance on the WIN (*N* = 1,205). Darker shades of gray and black are indicative of higher density of datapoints (PTA, pure tone average; PTA_SF_, standard frequency PTA; PTA_EHF_, extended high frequency PTA).

**Figure 3:**
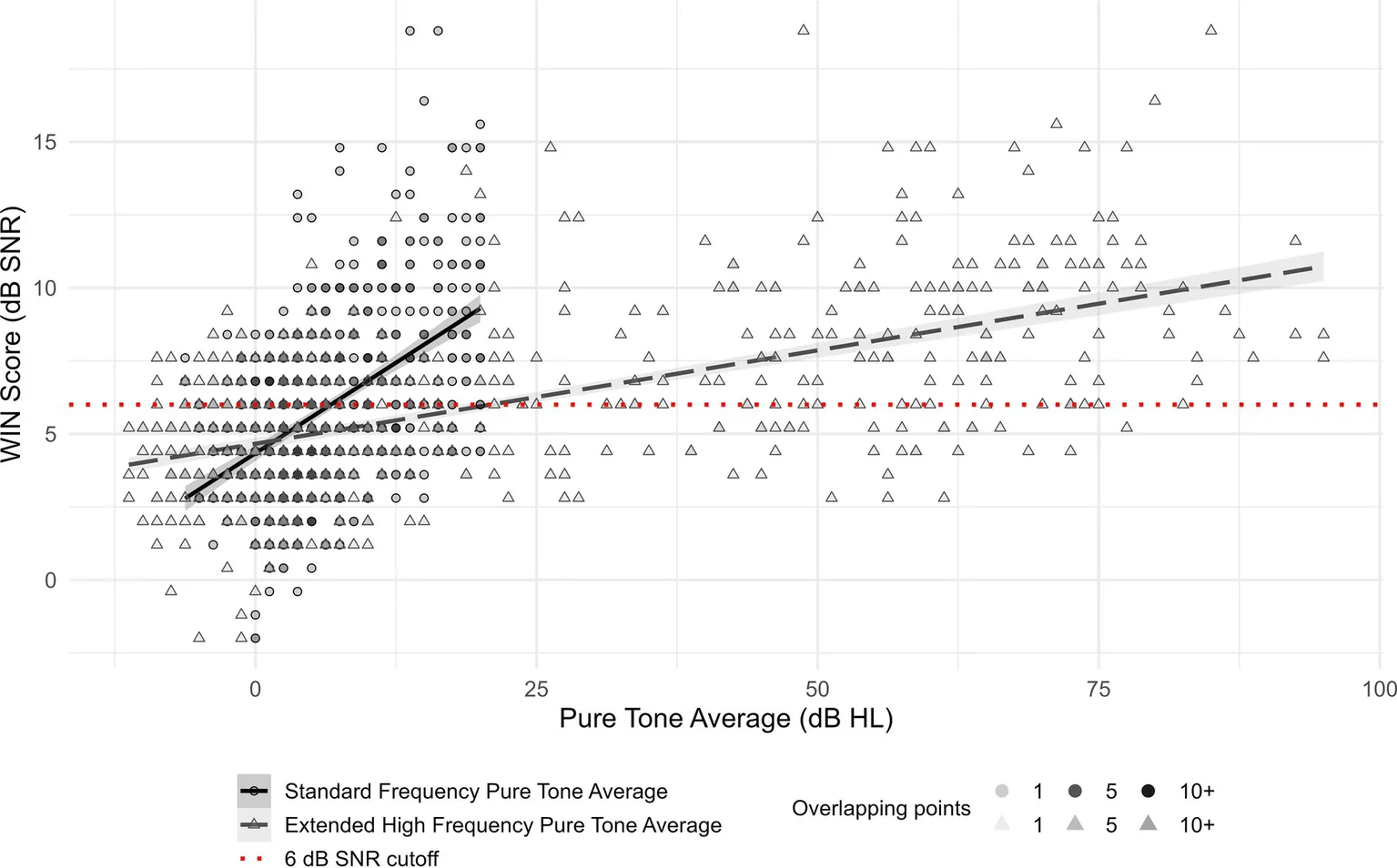
WIN performance as a function of PTA_SF_ (circles) and PTA_EHF_ (triangles) in normal hearing (PTA_SF_ ≤ 20 dB HL) listeners. The cutoff for normal speech-in-noise performance on the WIN (6 dB SNR) is indicated by the dashed red line. Darker shades of gray and black are indicative of higher density of datapoints. WIN performance was associated with hearing sensitivity in both the SF and EHF ranges, but more variation in performance can be attributed to PTA_EHF_ than PTA_SF_ in those with conventionally normal hearing. WIN performance in those with “normal hearing” had interpretations ranging from normal speech-in-noise performance to a severe speech-in-noise impairment (PTA, pure tone average; SF, standard frequency; EHF, extended high frequency; PTA_SF_, standard frequency PTA, PTA_EHF_, extended high frequency PTA, WIN, Words-in-Noise).

Step sizes in audiometric evaluations have been long established as 5 dB to provide enough variability to maintain sensitivity of the auditory system during testing and a narrow enough level difference to obtain a precise threshold measurement (Carhart and Jerger James, 1959). To further explore if there is an identifiable PTA_SF_ and PTA_EHF_ cutoff value where performance is abnormal, the normal hearing PTA_SF_ group was separated into the following 5-dB bins in Figure 4 (with number of ears indicated in parentheses): ≤ 0 (*N* = 113), 0 to ≤5 (*N* = 280), 5 to ≤10 (*N* = 145), 10 to ≤15 (*N* = 94), and 15 to ≤20 (*N* = 68) dB HL. As shown in Figure 4, the LME revealed statistically significant associations between WIN threshold and PTA_EHF_ in PTA_SF_ bins between >0 and <20 dB HL (0–5 dB HL: *r* = 0.28, *p* < 0.001; 5–10 dB HL: *r =* 0.41, *p* < 0.001; 10–15 dB HL: *r* = 0.33, *p* < 0.01).

**Figure 4:**
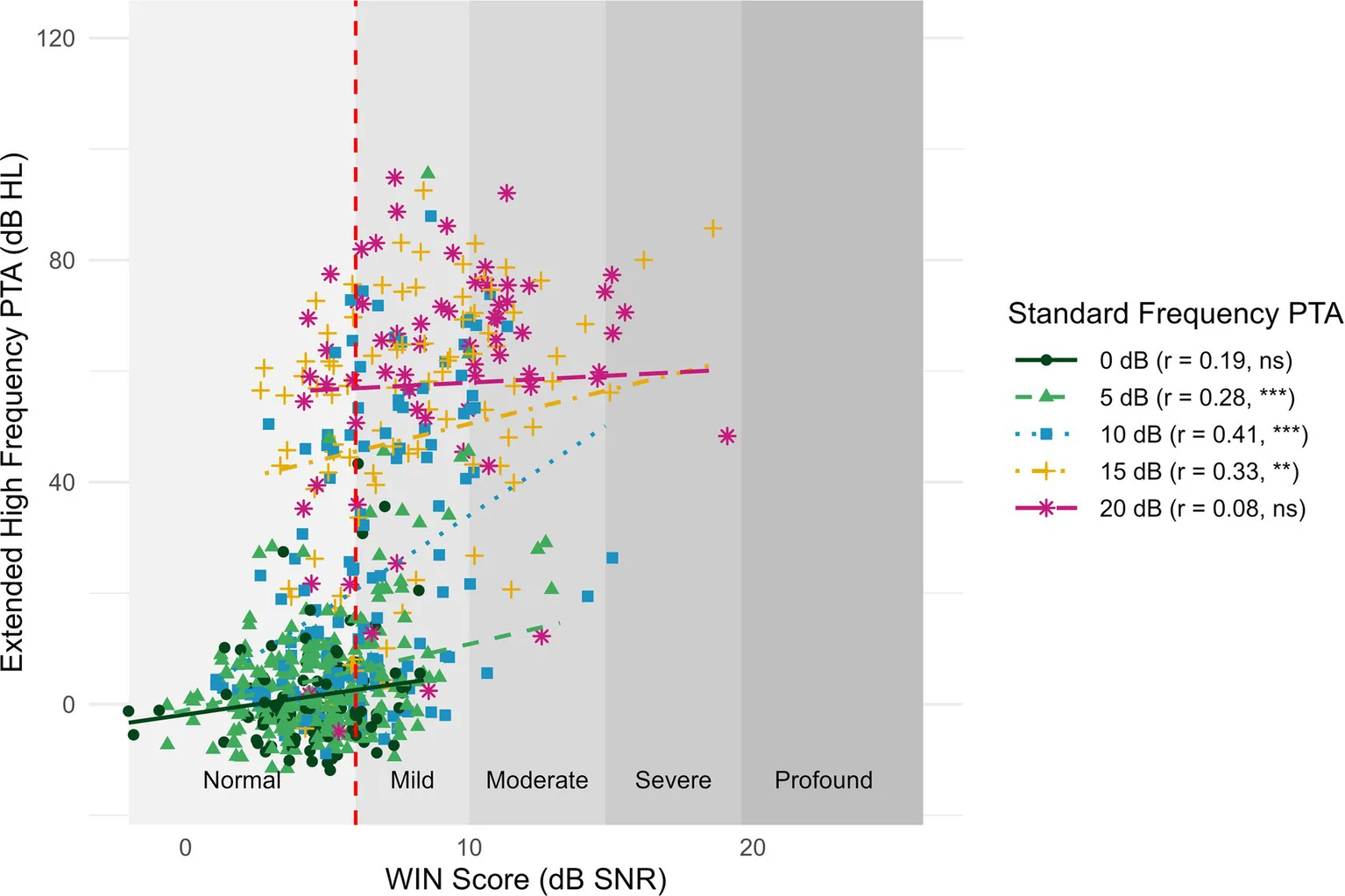
PTA_EHF_ in dB HL as a function of WIN Threshold in dB SNR. WIN threshold is separated into the following bins: PTA_SF_ ≤ 0 dB, 0–5 dB, 5–10 dB, 10–15 dB, and 15–20 dB. PTA_SF_ is parameterized by symbol, as indicated by the inset. A linear mixed effects model showed the PTA_EHF_ for PTA_SF_ bins 0–15 dB HL were significantly associated with WIN thresholds. The given *r* values are derived from effect sizes within the model (PTA, pure tone average; SF, standard frequency; EHF, extended high frequency; PTA_SF_, standard frequency PTA, PTA_EHF_, extended high frequency PTA; WIN, Words-in-Noise).

When we evaluated which PTA_SF_ bins were statistically different from 6 dB SNR, as seen in Figure 5, PTA_SF_ bins ≤0 and >0 to ≤5 dB HL had significantly better performance (*p* < 0.001). The >5 to ≤10 dB HL bin was not significantly different from the cutoff value (*p* = 0.29). Lastly, statistically significant abnormal WIN thresholds were observed beginning with the >10 to ≤15 dB-bin group and in all subsequently worse groups (*p* < 0.001; also see Supplementary Table 2).

**Figure 5:**
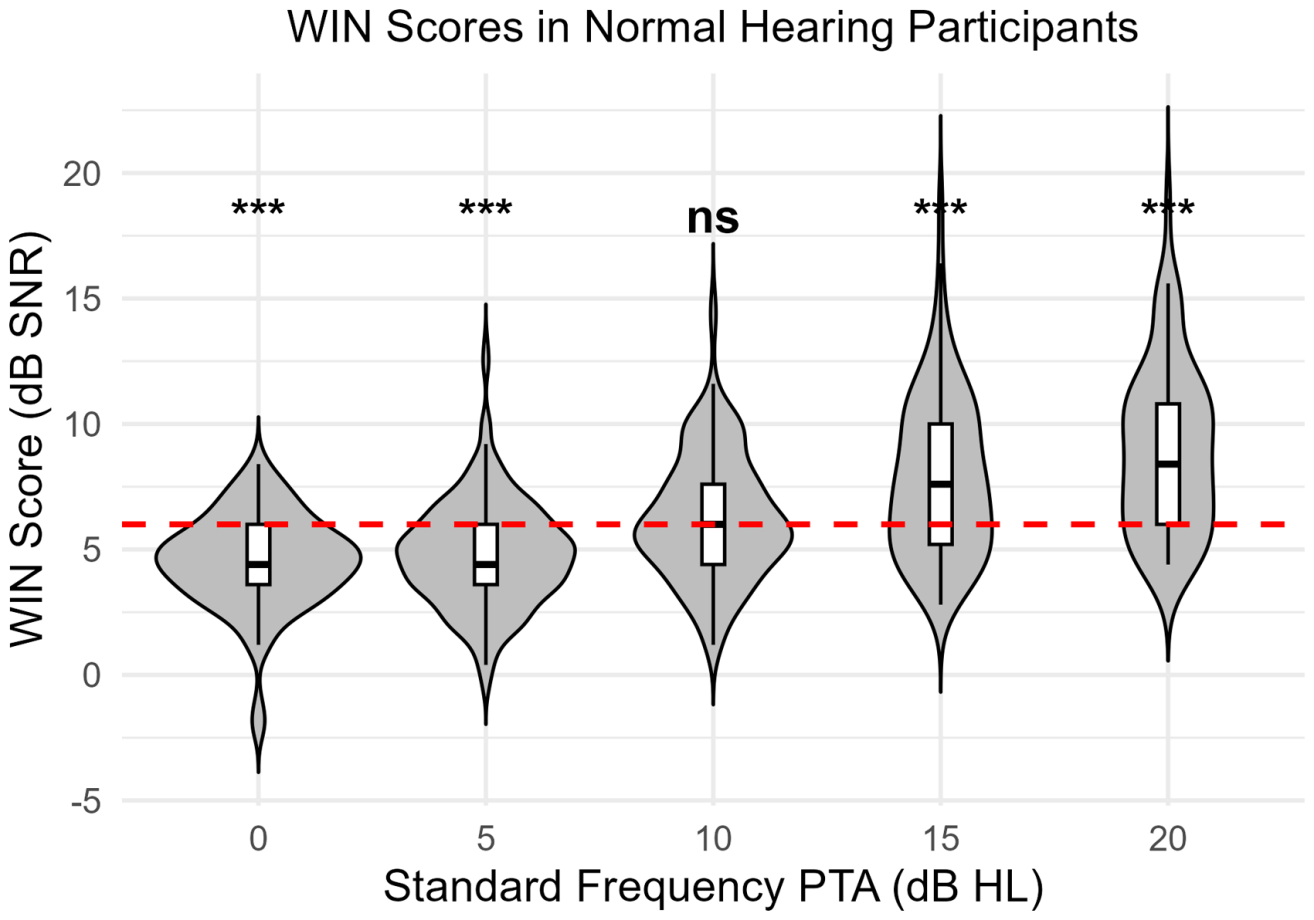
Participants were grouped by PTA_SF_ in 5 dB bins. Mixed linear models controlling for participant were computed to determine whether the mean WIN threshold of each PTA bin was statistically different from the 6 dB SNR cutoff value for normal speech-in-noise performance on the WIN. Detailed results are shown in Supplementary Table 2 (PTA, pure tone average; SF, standard frequency; EHF, extended high frequency; PTA_SF_, standard frequency PTA; WIN, Words-in-Noise).

As seen in Figure 6, the PTA_EHF_ was similarly broken into 5-dB bins from <−10 dB HL up to >90 to ≤95 dB HL. For PTA_EHF_, the <−10 to −10 and 85 to <90 dB HL bins did not fit the model due to the low N. As a result, a one-sample t-test was run for each of those bins. PTA_EHF_ bins > − 10 to ≤ 15 dB HL were significantly better than the 6-dB-SNR criterion. Bins >15 up to ≤ 40 dB HL were not significantly different from the normative cutoff, whereas the WIN thresholds in the PTA_EHF_ bins >40 dB HL and above were statistically poorer than the 6-dB-SNR cutoff value (also see Supplementary Table 3).

**Figure 6:**
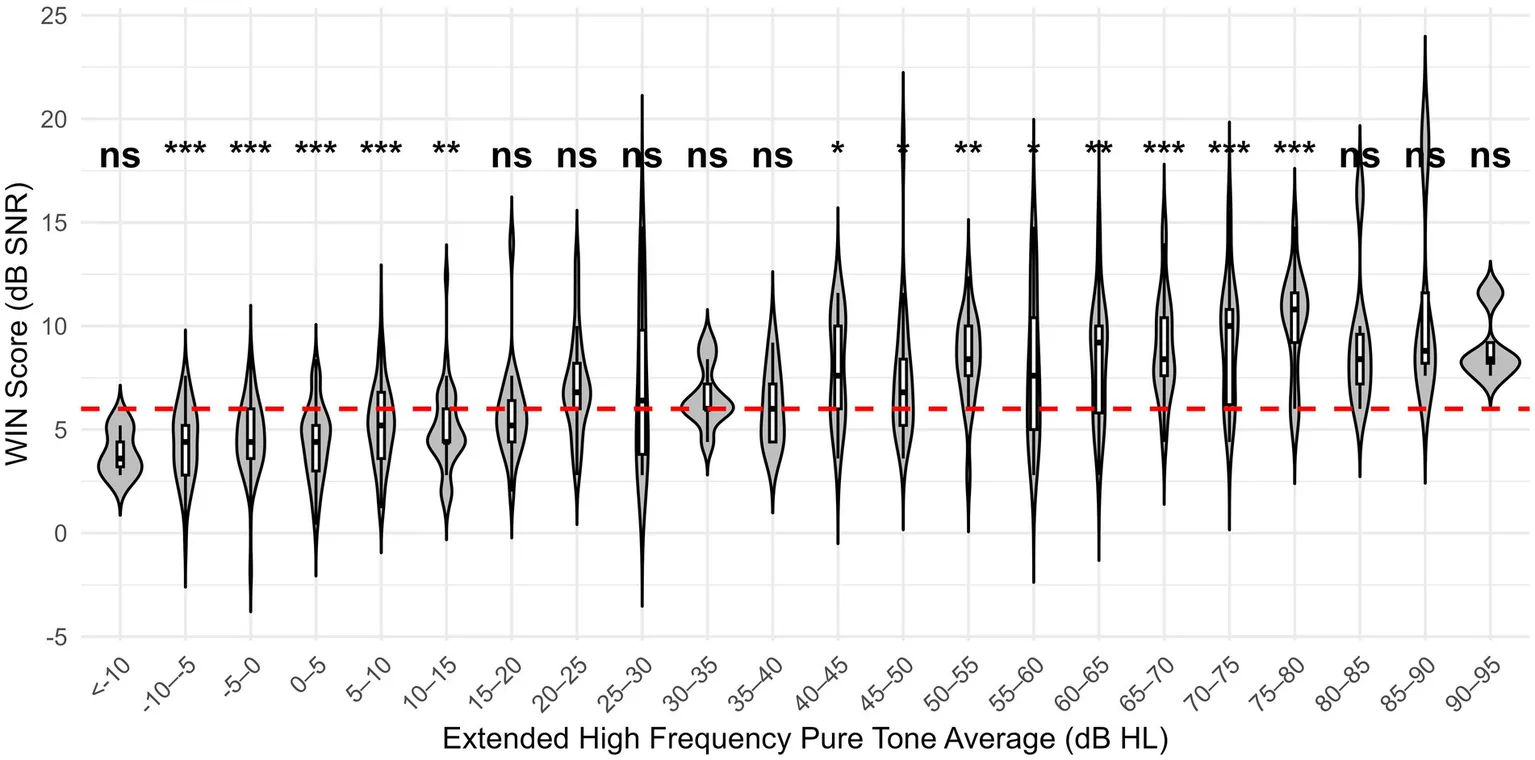
Participants were grouped by PTA_EHF_ in 5 dB bins. Mixed linear models were computed to evaluate whether the average WIN threshold for each PTA bin was significantly different from 6 dB SNR. Detailed results are shown in Supplementary Table 3 (PTA, pure tone average; EHF, extended high frequency; PTA_EHF_, extended high frequency PTA; WIN, Words-in-Noise).

Finally, because speech-in-noise deficits despite normal hearing thresholds are commonly seen in the literature but lack clinical guidelines, we explored the range of WIN performances within our normal hearing population. In Figure 7, we specifically assessed the relationship between EHF hearing and WIN performance using the top 10% (largest dB SNR, *N* = 81, mean = 11.6 dB SNR) and bottom 10% (smallest dB SNR, *N* = 98, mean = 2.06 dB SNR) of WIN thresholds within the NH group. The LME showed a significant difference in PTA_EHF_ between these the top (mean = 58.7 dB HL, SD = 18.3) and bottom (mean = 3.01 dB HL, SD = 11.8) WIN thresholds (*b* = 55.27, SE = 2.64, *t*(126.4) = 20.98, *p* < 0.001).

**Figure 7:**
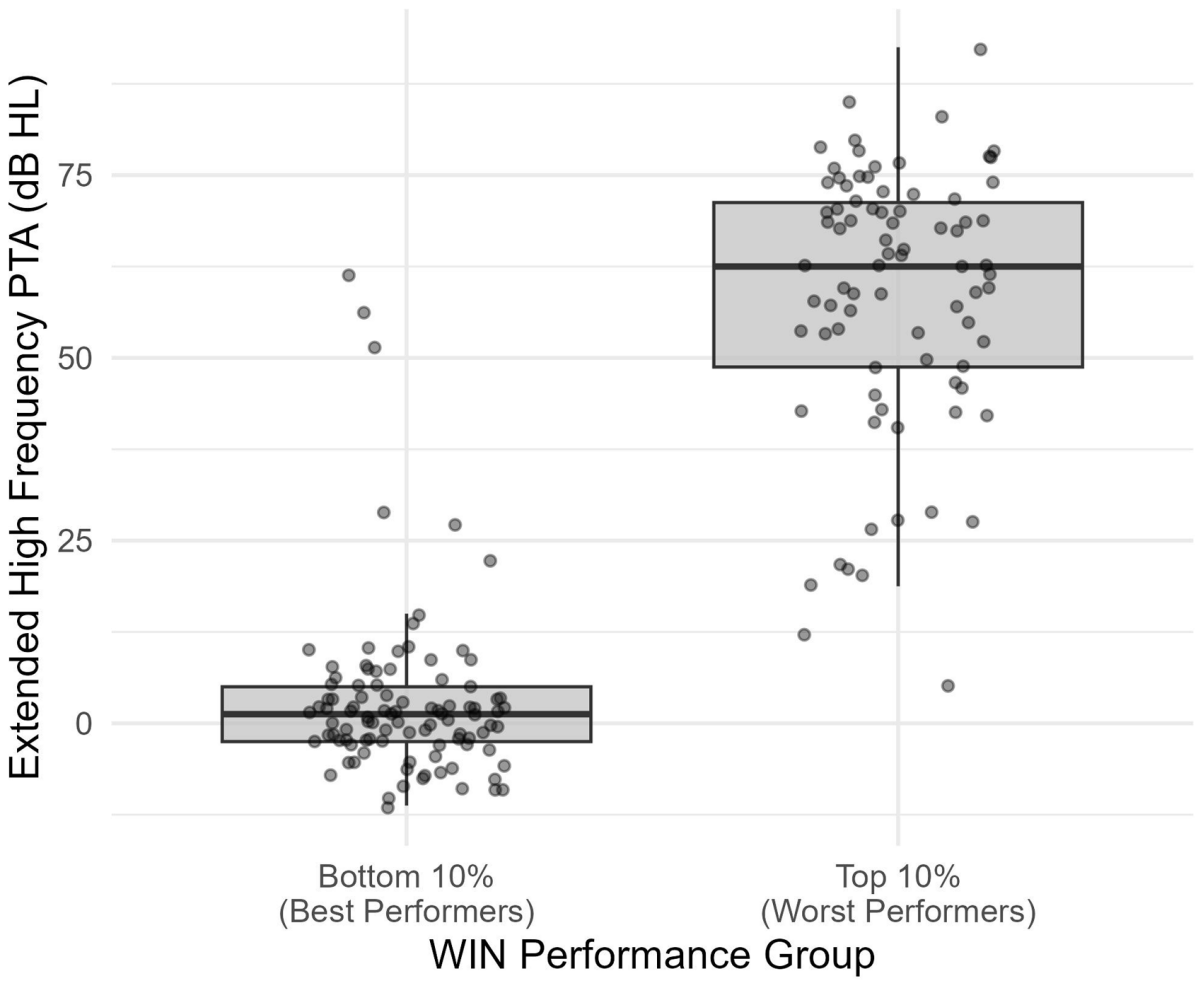
WIN performance was broken down into the top and bottom 10% of performance. We analyzed the PTA_EHF_ of each group. The best WIN performers had significantly better PTA_EHF_ than those with the worst WIN performance (PTA, pure tone average; EHF, extended high frequency; PTA_EHF_, extended high frequency PTA, WIN, Words-in-Noise).

### Sensation level

The NASEM report on outcome measures in audiology did not provide recommendations on presentation level for the WIN (National Academies of Sciences, Engineering, and Medicine, 2025). Our dataset includes WIN thresholds collected at various presentations levels across all hearing statuses, which results in participants being evaluated at various sensation levels (SL). To assess if presentation level affects performance on the WIN, we calculated both SL_SF_ and SL_EHF_ using the respective PTAs and present the data in Figure 8. Using a linear mixed effects model with a participant-level random intercept, we found WIN thresholds to be significantly affected by both SL_SF_ and SL_EHF_ (marginal R^2^ = 0.637). Accounting for between-participant variability increased the explained variance to 88.2% (conditional *R*^2^ = 0.882). SL_SF_ (*β* = −0.147, *t* = −15.64, *p* < 0.001) and SL_EHF_ (*β* = −0.043, *t* = −8.8, *p* < 0.001*)* were significant negative predictors, indicating that level of audibility can indeed affect performance.

**Figure 8:**
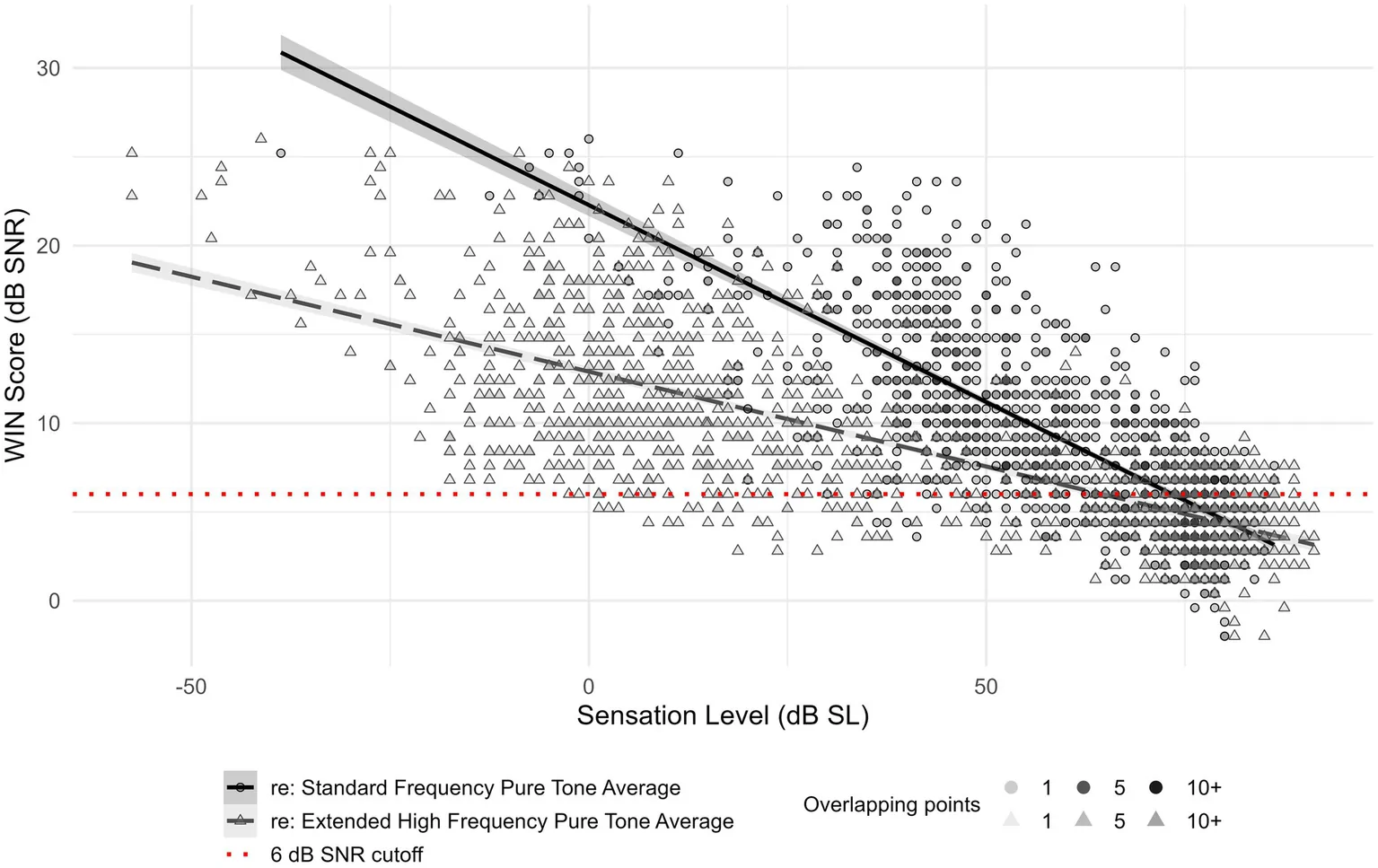
WIN performance as a function of sensation level (SL) calculated based on PTA_SF_ (circles) and PTA_EHF_ (triangles). SL was calculated as the difference between presentation level of the WIN and PTA for each frequency range. Increased SL in both the SF and EHF ranges was associated with improved performance indicating that level of audibility affects WIN performance. Darker shades of gray and black are indicative of higher density of datapoints (PTA, pure tone average; SF, standard frequency; EHF, extended high frequency; PTA_SF_, standard frequency PTA, PTA_EHF_, extended high frequency PTA, WIN, Words-in-Noise).

### Cognition

Prior literature has indicated that cognitive status could be a confounding variable for speech-in-noise performance (Dryden et al., 2017). Therefore, we evaluated the relative contributions of cognition as measured by the MoCA and PTA_EHF_ on speech-in-noise abilities and present the results in Figure 9. In our database, 524 participants were assessed using the MoCA. Because the MoCA is administered binaurally in the free field, WIN thresholds and PTAs were averaged across the left and right ear in this analysis. Our overall model was statistically significant, *F*(2, 521) = 393.9, *p* < 0.001, R^2^ = 0.602, indicating that the model explained 60.2% of the variance in WIN thresholds. EHF hearing sensitivity and MoCA score were significant predictors of WIN thresholds with EHF hearing sensitivity having a stronger effect, and PTA_EHF_ was a significant positive predictor of WIN threshold (*b* = 0.099, *SE* = 0.004, *t* = 25.39, *p* < 0.001), explaining 49.3% of variance after removing shared variance from MoCA scores, such that poorer EHF hearing sensitivity was associated with poorer WIN performance. The MoCA scores were also a significant negative predictor of WIN threshold (*b* = −0.286, *SE* = 0.066, *t* = −4.345, *p* < 0.001), explaining 1.4% of variance after removing shared variance with PTA_EHF_, indicating that better cognitive function was associated with better speech-in-noise performance. The remaining 9.5% of the model’s explained variance (*R*^2^ = 0.60) was shared between PTA_EHF_ and MoCA score.

**Figure 9:**
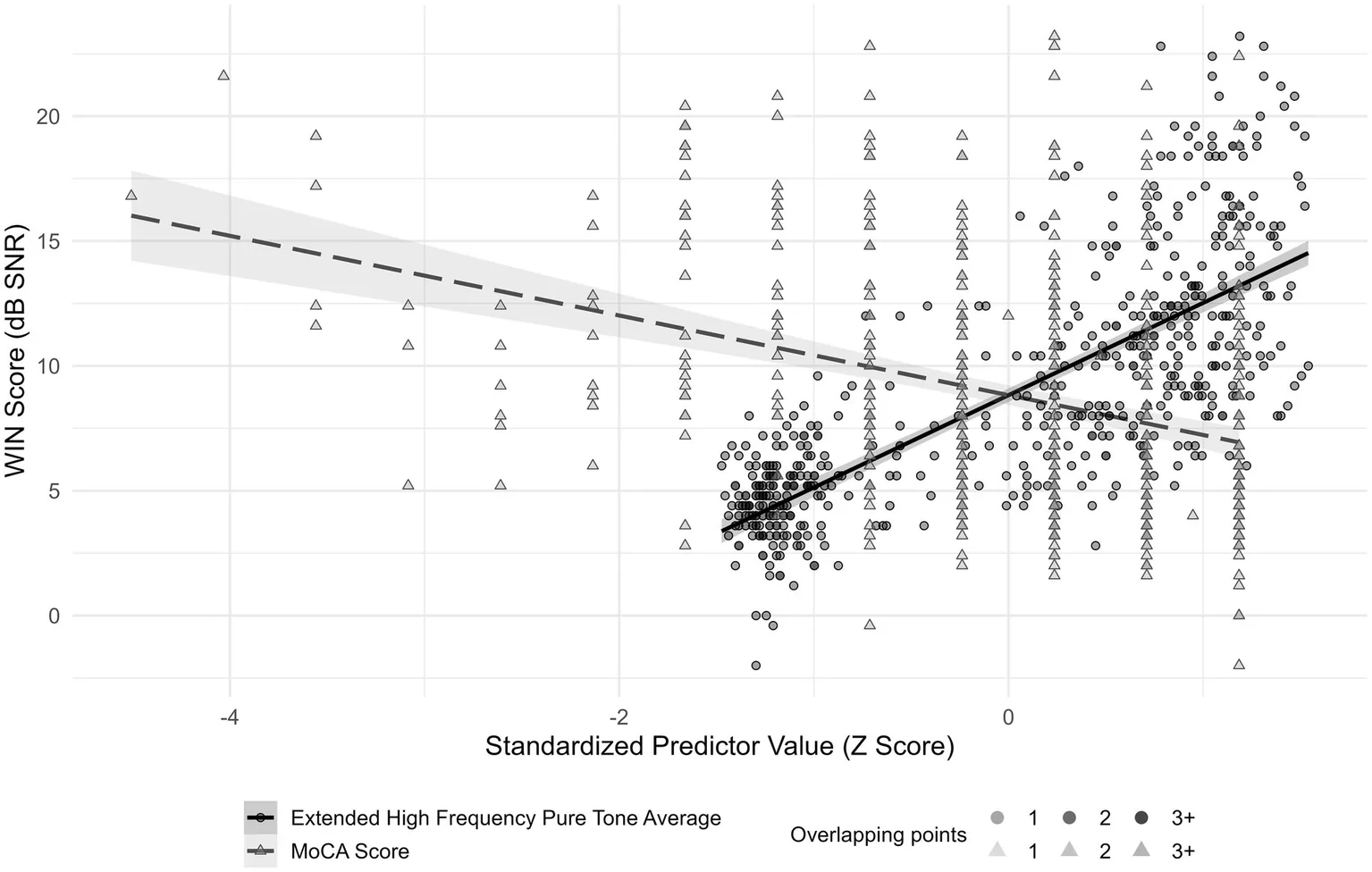
MoCA scores were collected and analyzed to assess EHF hearing sensitivity and cognition as predictors of WIN performance. WIN thresholds and PTA_EHF_ were averaged across ears due to MoCA instructions being delivered diotically. PTA_EHF_ and MOCA both have significant correlations with WIN thresholds. EHF_PTA_ is a stronger predictor of WIN thresholds. Multiple linear regression and semi-partial correlations were conducted to examine whether high-frequency hearing sensitivity (PTA_EHF_) and cognitive function (MoCA score) predicted WIN performance. PTA_EHF_ was a significant positive predictor and MoCA score was a significant negative predictor (PTA, pure tone average; PTA_SF_, standard frequency PTA, PTA_EHF_, extended high frequency PTA; WIN, Words-in-Noise; MocA, Montreal Cognitive Assessment).

### Age

Age is a complex variable in auditory research as it is highly correlated with degraded hearing sensitivity (Dorey et al., 2026) and cognition (Mamo et al., 2019). The results of the model using the 524 participants with MoCA scores indicate that PTA_EHF_ had the largest effect on WIN performance (*β* = 2.73, SE = 0.382, t = 7.142, *p* < 0.001). That is, participants with poorer EHF hearing sensitivity performed substantially worse on the WIN to a greater degree than age and cognitive status. Age had a small positive effect, indicating that older participants tend to have slightly worse speech-in-noise performance when controlling for hearing and cognition (*β* = 0.86, SE = 0.384, t = 2.242, *p* < 0.05), and MoCA scores had a small negative effect, indicating that participants with better cognitive scores tended to have slightly better performance on the WIN, after adjusting for hearing and age (*β* = −0.575, SE = 0.139, t = −4.137, *p* < 0.001).

### Sex

Sex differences were assessed for auditory and cognitive performance using a one-way multivariate analysis of variance (MANOVA). In Figure 10, sex differences were examined across WIN performance, MoCA scores, PTA_SF_, and PTA_EHF_. Figure 11 shows sex differences in WIN performance by decade of age. There was a multivariate effect of sex (Wilks’ λ = 0.97, *F*(4, 523) = 10.28, *p* < 0.001), in which males performed worse than females. Univariate analyses of variance (ANOVAs) were run for all variables, and results indicated that females had significantly better WIN performance (*F*(1, 526) = 10.28, *p* < 0.001; Figures 10a, 11), lower PTA_SF_ (*F*(1,526) = 26.808, *p* < 0.001; Figure 10b), and lower PTA_EHF_ (*F*(1,526) = 31.069, *p* < 0.001; Figure 10c) than males. Results from the analysis indicated that cognitive scores were lower in males than females overall (Figure 10d; *F*(1, 526) = 7.94, *p* < 0.01).

**Figure 10:**
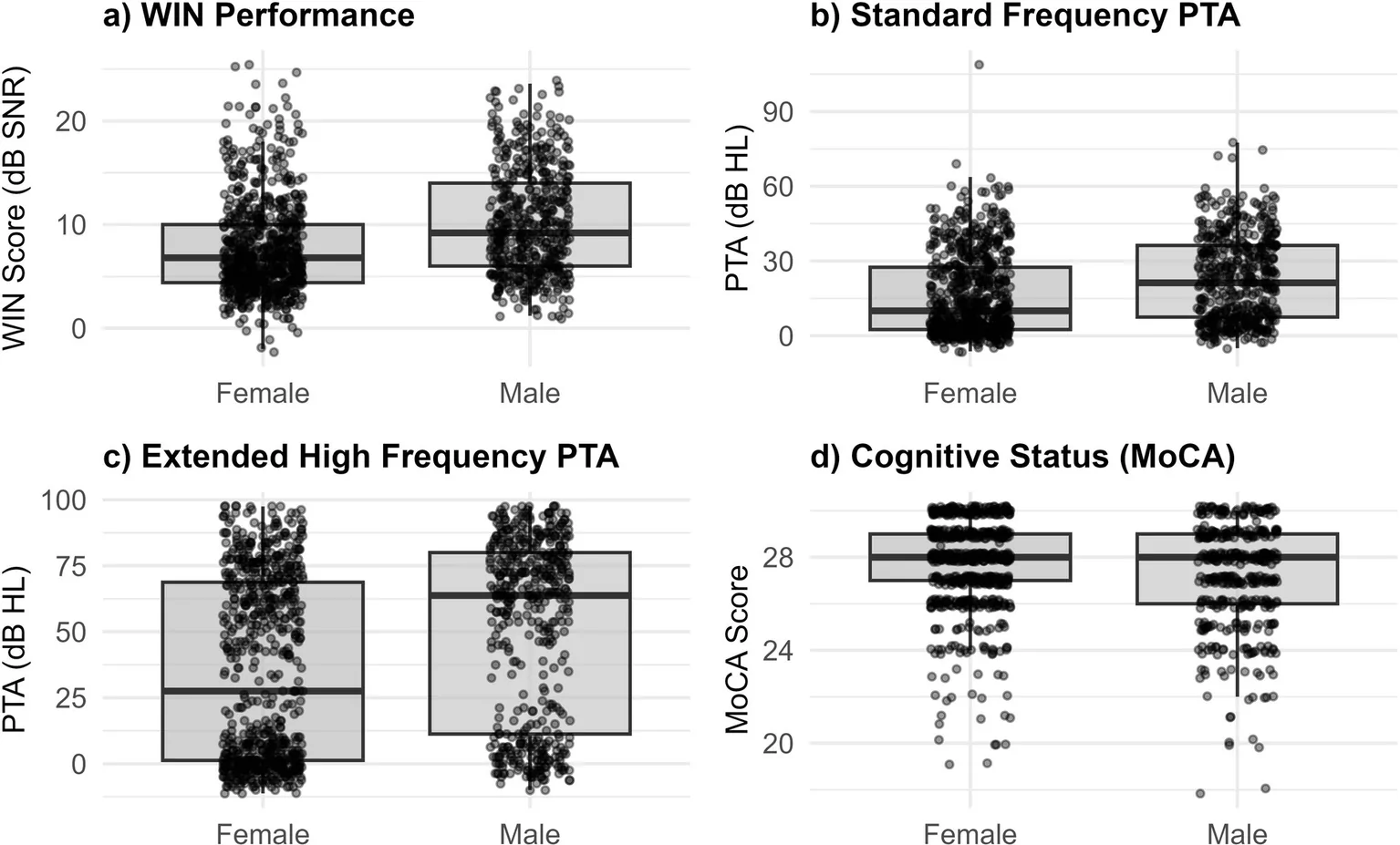
Sex differences were assessed for WIN performance **(a)**, PTA_SF_ **(b)**, PTA_EHF_ **(c)**, and MoCA scores **(d)**. Males scored significantly worse than females in all categories (PTA, pure tone average; SF, standard frequency; EHF, extended high frequency; PTA_SF_, standard frequency PTA; PTA_EHF_, extended high frequency PTA; WIN, Words-in-Noise; MocA: Montreal Cognitive Assessment).

**Figure 11:**
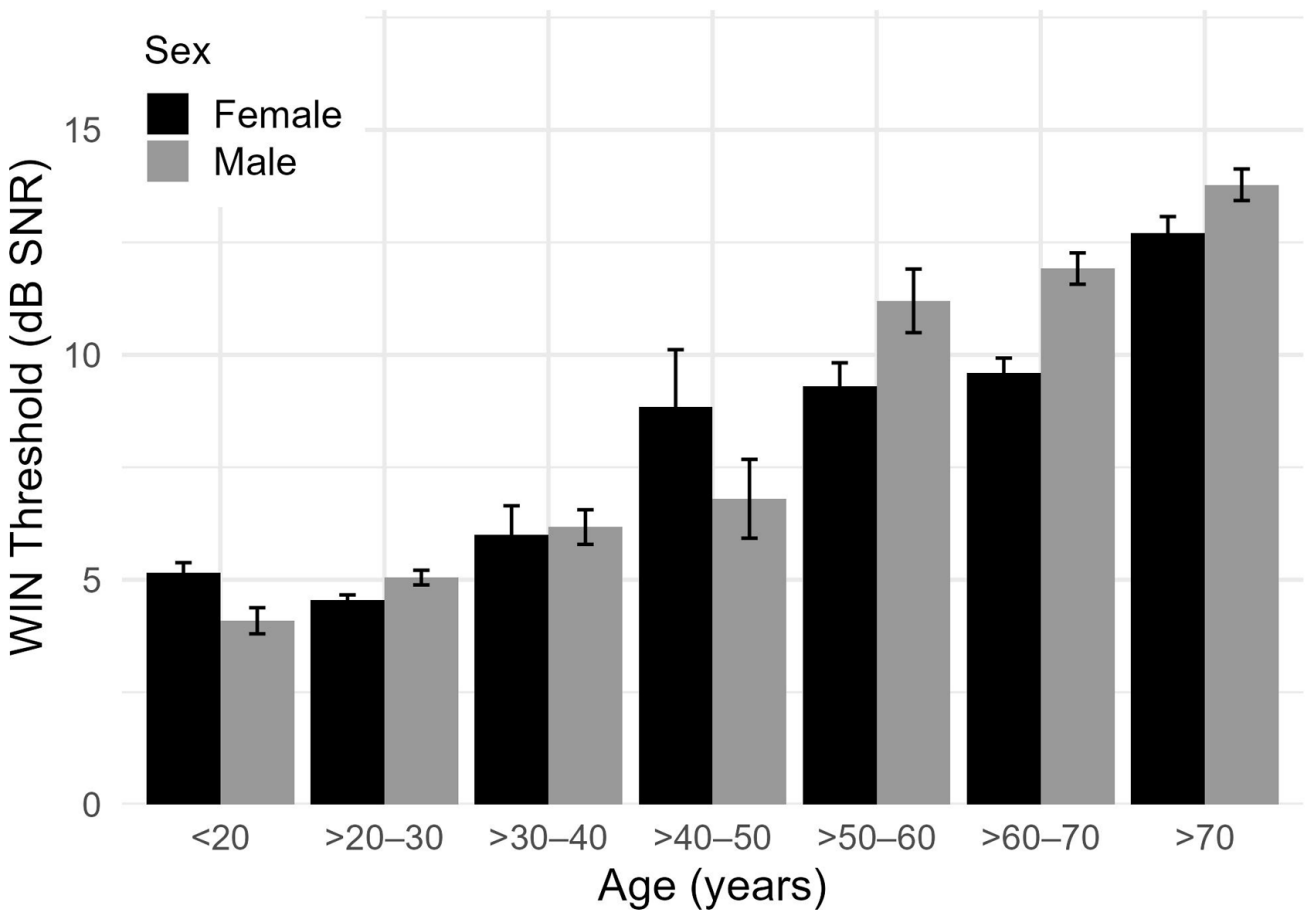
WIN thresholds as a function of age group (<20, >20 - ≤ 30, >30 - ≤ 40, >40 - ≤ 50, >50 - ≤ 60, >60 - ≤ 70, and >70 years) for females and males (WIN, Words-in-Noise).

## Discussion

Speech-in-noise difficulty is a primary complaint among many with various degrees of hearing sensitivity, even among those with normal or near normal hearing. Our findings support a relationship between elevated EHF thresholds and degraded speech-in-noise performance, independent of age and cognitive status, with males showing worse auditory and cognitive profiles than females. Impaired speech-in-noise performance, as measured by the WIN may serve as a functional indicator of normal vs. abnormal hearing sensitivity in the EHF range, and our analysis of the candidate range of EHF thresholds with impaired speech-in-noise performance could lead to establishment of a normative cutoff value for EHF hearing sensitivity.

Speech-in-noise performance, cognitive decline, and hearing loss have all shown associations in the literature. However, the nature of these relationships remains under debate, and the role of EHF hearing is relatively new to the discussion. In the present study, while both EHF hearing sensitivity and cognition were found to have a relationship with speech-in-noise performance, EHF hearing was the stronger predictor. This finding warrants further evaluation and discussion due to mixed results in previous work. Helfer et al. (2024) evaluated effects of EHF hearing on speech-in-noise performance (using the Revised Speech-in-Noise [SPIN] test; Kalikow et al., 1977; Stroop, 1935) and on cognition (using the Wisconsin Card Sorting Test and Color-Word Task Score; Kongs et al., 2000) and found mixed results. Though the authors found PTA_EHF_ was indeed correlated with perceived hearing difficulty, correlations with cognitive function varied between middle-aged and older populations and their data did not support a relationship between speech-in-noise performance and PTA_EHF_. Middle-aged participants had an association for increased effort related to their PTA_EHF_, but not performance on the cognitive tasks. The inverse was true in older adults, suggesting a complex and varied relationship between PTA_EHF_ and cognition. Additionally, their PTA_EHF_ was correlated with low context SPIN performance, but did not explain any additional variance when lower frequency PTAs were added into their model. The authors conclude more research is required to determine clinical relevance of EHF audiometry for older populations.

In contrast, other studies have supported the conclusion that there exists a relationship between speech-in-noise performance and EHF thresholds (e.g., Mishra et al., 2022b). Some key differences between Helfer et al. (2024), and other reports in the literature, including this one, are differing measures of speech-in-noise and cognitive performance, and importantly, the PTA_EHF_ in Helfer et al. extended only to 12 kHz. Alternatively, Waechter et al. (2022) found that EHF hearing sensitivity through 16 kHz was associated with cognitive performance, for example, and because our cognitive assessments were limited to the MOCA, instead of a full cognitive test battery, the debate may be unsettled. Future analysis including MoCA age-corrected norms could provide additional insight (Borland et al., 2017; Kistler-Fischbacher et al., 2025; Malek-Ahmadi et al., 2015), and because the WIN is also a monosyllabic word test perhaps associated more with sensitivity, future work might include a sentence level test that could have greater cognitive impact.

This is an especially important discussion in light of the recent *NASEM* report (National Academies of Sciences, Engineering, and Medicine, 2025), which chose the WIN as a recommended clinical outcome measure due to the strong development of the psychometric function, the test–retest reliability, and performance aligning with perceived difficulty, in contrast to other commercially available speech-in-noise tests. However, a standard presentation level was not recommended, and our data suggest that sensation level can influence WIN performance, making these recommendations perhaps premature and requiring further exploration, potentially involving analysis of a level series to assess various presentation levels (Figure 8).

It is possible that the WIN is more sensitive to peripheral compromise and subclinical deficits than other speech-in-noise tests (Wilson et al., 2007), especially in those with conventional normal or near normal hearing sensitivity. EHF hearing was associated with WIN thresholds in the full population assessed in the present study. This relationship is further supported by a significant difference in PTA_EHF_ between the top and bottom 10% of WIN performers, with the highest-performing group exhibiting better EHF hearing sensitivity. However, a subset of participants with normal hearing in the conventional audiometric range (PTA_SF_ < 25 dB HL) had abnormal WIN thresholds, despite being classified in conventional clinical practice as audiometrically “normal,” implying normal performance. In our dataset, significant speech-in-noise deficits, as determined by the WIN, were associated with participants with PTA_SF_ as low as 10 to 15 dB HL, further supporting the evidence of speech-in-noise deficits in patients below even the most conservative of audiometric normal cutoff values. While it is possible this evidence supports an inflated cutoff value of 25 dB HL in the standard frequency range for normal hearing (Gatlin and Dhar, 2021), this effect may be in part due to worsening of thresholds in the EHF range. When we look at the same relationship between our PTA_EHF_ groups, mean WIN thresholds with impaired performance (i.e., worse than the 6-dB SNR cutoff value) were associated with PTA_EHF_ as low as 15 dB HL, with statistical significance reached at 40 dB HL.

This finding is particularly of note since the WIN stimuli have limited spectral energy in the EHF range (Supplementary Figure 1). While spectral energy extends to 10 kHz, the magnitude of the target signal decreases significantly beyond 8 kHz. This is a common limitation in conventional speech materials (Monson and Buss, 2022), and the energy can be further limited by the transducer used to present the stimuli (Smull et al., 2019). Despite the limited EHF spectral information in the WIN, performance on this test was nevertheless associated with EHF hearing sensitivity, indicating that mechanisms associated with encoding EHF signals may result in a degraded signal affecting lower frequency regions that are more classically associated with speech understanding. Our findings further support other literature showing that damage in the EHF range is associated with subclinical atypical function in the conventional range. Saade et al. (2024) found that 62.5% of their clinical population with normal hearing in the conventional range and elevated EHF thresholds had hearing complaints. Mishra et al. (2022b) found that poorer EHF thresholds were associated with higher SRTs in noise in pediatric population, and similarly, Motlagh Zadeh et al. (2019) found an association between EHF hearing and performance on the Digits in Noise test with a low-pass filter cutoff value of 8 kHz, which also aligned with participant self-reported hearing status.

One potential explanation for the association between EHF sensitivity and speech-in-noise performance involves changes in cochlear mechanics and/or supra-threshold neural encoding resulting from cochlear damage. Aryal and Mishra (2025) found that better EHF thresholds were associated with sharper cochlear frequency selectivity. Broadened frequency tuning curves in participants with elevated EHF thresholds may result in subclinical functional deficits, such as impaired speech perception, especially in the presence of background noise. Significant work in otoacoustic emissions has shown neural encoding and tuning changes in the standard frequency range as low as 1 kHz in both adult and pediatric populations (Aryal and Mishra, 2025; Hunter et al., 2021; Mishra et al., 2024; Mishra et al., 2022a, 2022b). Additional etiology may be due to disruptions of the voltage gradient where potassium ion channels may be compromised through the entire cochlea, stressing the basal end more than mid frequency structures, leading to downstream effects in signaling (Peixoto Pinheiro et al., 2021; Rías et al., 2025; Zdebik et al., 2009). This is particularly relevant given findings that hair cell loss post-mortem is significantly correlated to in-vivo audiograms, not other associated tissue in the inner ear (Wu et al., 2020).

Irrespective of the mechanisms related to changes in EHF sensitivity and its demonstrated association with speech-in-noise performance, addition of EHF thresholds to a standard clinical evaluation could provide clinical utility and insight. Impaired speech perception in noisy environments despite normal, or near normal, thresholds in the conventional audiometric range is a common patient complaint in outpatient audiology clinics (Bharadwaj and Shinn-Cunningham, 2021; Tremblay et al., 2015). Our data suggest that elevation in EHF thresholds could account for some of the variance in WIN thresholds in this population. In our full participant population, including ears with traditionally defined hearing loss, the associations were similar between PTA_SF_ and PTA_EHF_ and WIN thresholds. However, when all participants in our hearing loss group were removed, PTA_SF_ only explained 10.4% of the variance, and PTA_EHF_ was a stronger predictor of WIN performance in normal hearing listeners. Therefore, EHF threshold measurement could be predictive of subclinical suprathreshold deficits, which is of pertinent diagnostic relevance in clinical care.

Patients with impaired EHF hearing sensitivity and otherwise “normal” hearing may also benefit from amplification. These individuals would be classified as having “subclinical” hearing loss and would be hearing aid candidates (Dragon et al., 2022; Frisby et al., 2024; Singh and Doherty, 2020). Should damage to the basal end of the cochlea be disrupting cochlear processes of the mid frequency range as theorized, providing mid to high frequency amplification could lessen perceived and measurable functional deficits, despite modern hearing aids not amplifying through the full EHF range. Additionally, hearing aid gain prescription based on the recent release of NAL-NL3 (National Acoustic Laboratories, 2025) now includes consideration for normal and near-normal hearing thresholds, allowing for improved hearing aid fittings in this population. Evaluating the use of the WIN as an outcome measure for hearing aid fitting as recommended by the NASEM report (National Academies of Sciences, Engineering, and Medicine, 2025), specifically in this population, may provide additional insight to the routine clinical utility of both EHF threshold measurement and sensitivity of the WIN for subclinical cochlear dysfunction.

One significant limitation preventing routine implementation of EHF threshold measurement in standard clinical practice is the establishment of a standard cutoff value defining normal vs. abnormal hearing sensitivity. This idea has been explored in depth in the literature with many reference datasets available (Dorey et al., 2026; Green et al., 1987; Rodríguez Valiente et al., 2014; Shahnaz and Ciocca, 2026; Stelmachowicz et al., 1989; Stenklev and Laukli, 2004), yet a clinically applicable cutoff value has not been determined, and functional performance has been mostly absent from the conversation. While much of the literature has analyzed normative hearing sensitivity in the EHF range by age, to establish a true cutoff value, functional performance is important to consider for the disease vs. absence of disease model that informs clinical decision making. In a similar light to the work which considered performance when establishing pediatric norms in the standard frequency range (Northern and Downs, 2002), our data suggest there is a candidate range in which EHF hearing sensitivity results in abnormal speech-in-noise performance (Figure 6).

In conclusion, worse EHF hearing sensitivity is associated with degraded speech-in-noise performance in both populations with hearing loss and those associated with normal hearing thresholds. Our data suggest that EHF hearing sensitivity has an even stronger association with speech-in-noise performance than cognitive status, although this is an area that requires further analysis with a complete neurocognitive test battery. Prospective research with more comprehensive cognitive work is necessary to establish a causal relationship. As a result, EHF threshold measurement could provide clinical utility in audiologic evaluations in all patients, with impact of this information strengthened after a clinical cutoff value has been established in the field.

## Data Availability

The raw data supporting the conclusions of this article will be made available by the authors, without undue reservation.
