# Supplementary material for: Relationships between extended high frequency hearing, cognition, and speech-in-noise performance
Source: Front Neurosci. 2026 Oct 20;20:1933436. doi: 10.3389/fnins.2026.1933436 (PMC13727533; doi:10.3389/fnins.2026.1933436)
Supplement: Supplementary file 1 [file Table_1.docx]

Supplemental Table 1. Reported Level at maximum output of the audiometer by frequency. No response thresholds were reported as 5 dB above the maximum output of the audiometer at that frequency.

| **Frequency** | **Maximum Output Level (db HL)** | **Reported Level (dB HL)** | **RETSPL** |
| --- | --- | --- | --- |
| 250 | 110 | 115 | 18 |
| 500 | 115 | 120 | 11 |
| 1000 | 120 | 125 | 5.5 |
| 1500 | 115 | 120 | 5.5 |
| 2000 | 115 | 120 | 4.5 |
| 3000 | 115 | 120 | 2.5 |
| 4000 | 115 | 120 | 9.5 |
| 6000 | 105 | 110 | 17 |
| 8000 | 100 | 105 | 17.5 |
| 9000 | 100 | 105 | 19 |
| 10000 | 100 | 105 | 22 |
| 11200 | 95 | 100 | 23 |
| 12500 | 90 | 95 | 27.5 |
| 14000 | 80 | 85 | 35 |
| 16000 | 60 | 65 | 56 |

Supplemental Table 2: PTA_SF_ results of linear mixed effects model controlling for participant. PTA_SF_ bins <0 to 5 dB HL were significantly better and PTA_SF_ bins 10 to 20 dB HL than the 6 dB SNR cutoff threshold.

| PTA_SF_ bin (dB HL) | N (ears) | N (participants) | Mean WIN Score (dB SNR) | SE | *t*-statistic | *p* value |
| --- | --- | --- | --- | --- | --- | --- |
| < 0 | 113 | 85 | 4.60 | 0.20 | -7.13 | <0.001 |
| 0 – 5 | 280 | 190 | 4.86 | 0.15 | -7.13 | <0.001 |
| 5 – 10 | 145 | 110 | 6.26 | 0.24 | 1.07 | 0.29 |
| 10 – 15 | 94 | 71 | 7.81 | 0.37 | 4.96 | <0.001 |
| 15 – 20 | 68 | 51 | 9.02 | 0.45 | 6.68 | <0.001 |

Supplemental Table 3: Linear mixed effects models holding participant as a constant were computed for each PTA_EHF_ bin to determine whether WIN scores were significantly different from the established normative cutoff of 6 dB SNR. The <-10 to -10 and 85 to <90 dB HL bins were run using a t-test since the N was not sufficient to fit the model. Relative to the 6-dB normative cutoff, WIN scores were significantly lower for PTA_EHF_ bins <20 dB HL, were not significantly different for bins from 15 to 39 dB HL and were significantly higher for bins ≥40 dB HL.

| PTA_EHF_ bin (dB HL) | N (ears) | N (participants) | Mean WIN Score (dB SNR) | SE | *t*-statistic | *p* value |
| --- | --- | --- | --- | --- | --- | --- |
| <-10 | 3 | 3 | 3.87 | 0.71 | -3.02 | 0.09 |
| -10 – -5 | 49 | 39 | 4.34 | 0.27 | -6.20 | <0.001 |
| -5 – 0 | 139 | 105 | 4.66 | 0.17 | -7.74 | <0.001 |
| 0 – 5 | 130 | 106 | 4.42 | 0.18 | -8.98 | <0.001 |
| 5 – 10 | 90 | 74 | 5.18 | 0.22 | -3.70 | <0.001 |
| 10 – 15 | 42 | 36 | 5.02 | 0.34 | -2.88 | 0.007 |
| 15 – 20 | 19 | 18 | 5.80 | 0.59 | -0.34 | 0.74 |
| 20 – 25 | 22 | 18 | 7.05 | 0.53 | 2.01 | 0.06 |
| 25 – 30 | 14 | 12 | 7.07 | 1.12 | 0.96 | 0.36 |
| 30 – 35 | 8 | 7 | 6.54 | 0.55 | 0.99 | 0.36 |
| 35 – 40 | 7 | 6 | 6.23 | 0.77 | 0.29 | 0.78 |
| 40 – 45 | 14 | 12 | 7.97 | 0.76 | 2.58 | 0.03 |
| 45 – 50 | 23 | 21 | 7.56 | 0.73 | 2.15 | 0.04 |
| 50 – 55 | 17 | 15 | 8.32 | 0.60 | 3.86 | 0.002 |
| 55 – 60 | 24 | 18 | 7.96 | 0.82 | 2.39 | 0.03 |
| 60 – 65 | 24 | 18 | 8.33 | 0.68 | 3.42 | 0.003 |
| 65 – 70 | 23 | 22 | 9.07 | 0.55 | 5.56 | <0.001 |
| 70 - 75 | 22 | 19 | 9.41 | 0.67 | 5.09 | <0.001 |
| 75 – 80 | 15 | 13 | 9.97 | 0.76 | 5.26 | <0.001 |
| 80 – 85 | 7 | 6 | 9.66 | 1.44 | 2.54 | 0.051 |
| 85 – 90 | 4 | 4 | 11 | 2.62 | 1.91 | 0.15 |
| 90 - 95 | 4 | 3 | 9.20 | 1.0 | 3.21 | 0.09 |

Supplemental Figure 1: Long-term average spectrum (LTAS) of each target speaker for all word lists of the WIN at 24 dB SNR. There is spectral information through 10 kHz, but there is a reduction in magnitude between 8 and 10 kHz.
